# The Fibrotic Substrate in Persistent Atrial Fibrillation Patients: Comparison Between Predictions From Computational Modeling and Measurements From Focal Impulse and Rotor Mapping

**DOI:** 10.3389/fphys.2018.01151

**Published:** 2018-08-29

**Authors:** Patrick M. Boyle, Joe B. Hakim, Sohail Zahid, William H. Franceschi, Michael J. Murphy, Adityo Prakosa, Konstantinos N. Aronis, Tarek Zghaib, Muhammed Balouch, Esra G. Ipek, Jonathan Chrispin, Ronald D. Berger, Hiroshi Ashikaga, Joseph E. Marine, Hugh Calkins, Saman Nazarian, David D. Spragg, Natalia A. Trayanova

**Affiliations:** ^1^Department of Biomedical Engineering, Institute for Computational Medicine, Johns Hopkins University, Baltimore, MD, United States; ^2^Department of Cardiology, Johns Hopkins Hospital, Baltimore, MD, United States; ^3^Penn Heart & Vascular Center, University of Pennsylvania, Philadelphia, PA, United States

**Keywords:** atrial fibrillation, re-entrant drivers, fibrotic remodeling, ablation, computational modeling, intracardiac electroanatomic mapping

## Abstract

Focal impulse and rotor mapping (FIRM) involves intracardiac detection and catheter ablation of re-entrant drivers (RDs), some of which may contribute to arrhythmia perpetuation in persistent atrial fibrillation (PsAF). Patient-specific computational models derived from late gadolinium-enhanced magnetic resonance imaging (LGE-MRI) has the potential to non-invasively identify all areas of the fibrotic substrate where RDs could potentially be sustained, including locations where RDs may not manifest during mapped AF episodes. The objective of this study was to carry out multi-modal assessment of the arrhythmogenic propensity of the fibrotic substrate in PsAF patients by comparing locations of RD-harboring regions found in simulations and detected by FIRM (RD_sim_ and RD_FIRM_) and analyze implications for ablation strategies predicated on targeting RDs. For 11 PsAF patients who underwent pre-procedure LGE-MRI and FIRM-guided ablation, we retrospectively simulated AF in individualized atrial models, with geometry and fibrosis distribution reconstructed from pre-ablation LGE-MRI scans, and identified RD_sim_ sites. Regions harboring RD_sim_ and RD_FIRM_ were compared. RD_sim_ were found in 38 atrial regions (median [inter-quartile range (IQR)] = 4 [3; 4] per model). RD_FIRM_ were identified and subsequently ablated in 24 atrial regions (2 [1; 3] per patient), which was significantly fewer than the number of RD_sim_-harboring regions in corresponding models (*p* < 0.05). Computational modeling predicted RD_sim_ in 20 of 24 (83%) atrial regions identified as RD_FIRM_-harboring during clinical mapping. In a large number of cases, we uncovered RD_sim_-harboring regions in which RD_FIRM_ were never observed (18/22 regions that differed between the two modalities; 82%); we termed such cases “latent” RD_sim_ sites. During follow-up (230 [180; 326] days), AF recurrence occurred in 7/11 (64%) individuals. Interestingly, latent RD_sim_ sites were observed in all seven computational models corresponding to patients who experienced recurrent AF (2 [2; 2] per patient); in contrast, latent RD_sim_ sites were only discovered in two of four patients who were free from AF during follow-up (0.5 [0; 1.5] per patient; *p* < 0.05 vs. patients with AF recurrence). We conclude that substrate-based ablation based on computational modeling could improve outcomes.

**GRAPHICAL ABSTRACT:**
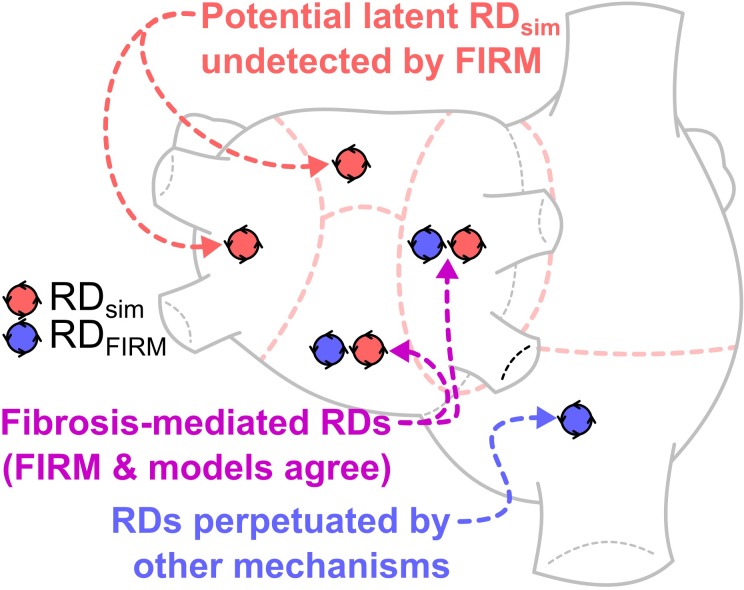
This study compares locations of re-entrant driver (RD)-harboring regions identified by intracardiac focal impulse and rotor mapping (FIRM) during catheter ablation procedures in persistent atrial fibrillation (PsAF) patients, and those identified by atrial computational simulations based on fibrosis imaging in the same patients. The finding of atrial regions in which both FIRM and simulations detected RDs (*purple*) reinforces a key understanding, namely that PsAF is in part driven by fibrosis-mediated mechanisms. The results of our analysis support the notion that ablation therapy based on clinical RD mapping is limited by the prevalence of latent RD sites (*red*), where the fibrotic substrate for RD perpetuation exists but re-entrant activity never manifests during the mapped AF episode(s). RD-harboring regions detected by FIRM but not in simulations (*blue*), indicating AF episodes perpetuated by mechanisms other than the fibrotic substrate, were not frequently seen in this cohort. This analysis suggests that substrate-based ablation based on computational modeling could improve outcomes.

## Introduction

Atrial fibrillation (AF) affects up to 2% of the population, making it the most prevalent sustained arrhythmia (Andrade et al., 2014). Pulmonary vein isolation (PVI) via catheter ablation can effectively treat some forms of AF (Haissaguerre et al., 1998; Calkins et al., 2017), but recurrence rates remain unacceptably high (40–60%) in patients with persistent AF (PsAF) (Verma et al., 2015). A potential explanation for the relative ineffectiveness of ablation in these patients is the fact that PsAF is often associated with atrial fibrosis, establishing a substrate for which the arrhythmogenic propensity is beyond the area affected by PVI (Burstein and Nattel, 2008; Nattel et al., 2008; Yue et al., 2011). Thus, new methodologies are needed to accurately identify the ablation targets in the fibrotic substrate.

One approach used in recent studies is focal impulse and rotor mapping (FIRM), one aim of which is to determine the locations of the re-entrant drivers (RDs; i.e., rotors) that contribute to AF perpetuation by interpreting intracardiac electrogram signals from multi-electrode basket catheters inserted in the atria during ablation procedures; identified RD sites are then targeted for ablation (Narayan et al., 2012b, 2014). One potential explanation for the limited ability of FIRM-guided ablation to achieve freedom from AF in some cases (Mohanty et al., 2018) is the failure to modify the arrhythmogenic substrate sufficiently to eliminate its capacity to sustain RDs. This notion is supported by evidence from some studies that AF may be sustained by persistent RDs at different locations in cases of failed ablation (Lalani et al., 2016; Boyle et al., 2018). Notably, FIRM also aims to identify sources of triggered excitation (i.e., focal impulses) but these are beyond the scope of the present investigation.

Simulations conducted in patient-specific computational models reconstructed from late gadolinium-enhanced magnetic resonance imaging (LGE-MRI) scans have recently been used to develop insights into the perpetuation and ablation of PsAF in patients with atrial fibrosis (McDowell et al., 2012; Trayanova, 2014; Boyle et al., 2016, 2017; Zahid et al., 2016a). Our work using such models has identified specific spatial patterns of fibrotic tissue that promote AF perpetuation (Zahid et al., 2016a) and these findings are corroborated by clinical evidence (Cochet et al., 2018). Also, in a study complementary to the present work, we compared rotor harboring regions, as characterized by pre-ablation non-invasive electrocardiographic imaging (ECGI), with those predicted in patient-specific models reconstructed from LGE-MRI scans of the same patients (Boyle et al., 2018). These studies all pointed to a key advantage of the computational approach to fibrotic substrate characterization: namely, simulations are capable of identifying latent rotor sites that may not manifest during clinical mapping.

The aim of this study was to carry out multi-modal assessment of pro-arrhythmic properties in the fibrotic substrate in PsAF patients by comparing RD-harboring regions found in simulations and detected by FIRM (RD_sim_ and RD_FIRM_) and analyze implications for ablation strategies predicated on targeting rotors. We retrospectively conducted simulations in personalized atrial models reconstructed from pre-procedure MRI scans for 11 PsAF patients who underwent FIRM-guided ablation at Johns Hopkins Hospital. Our study offers further insights into the importance of latent RD_sim_ sites in the fibrotic atria, which have implications for improving long-term outcomes of PsAF ablation procedures.

## Materials and Methods

### Persistent AF Patient Cohort

Eleven patients with PsAF who underwent pre-ablation LGE-MRI and FIRM as an adjunct to PVI in 2015 were included in this study. All 11 patients have been included in previous studies by our group (Chrispin et al., 2016; Balouch et al., 2017); the other patients included in those previous studies could not be included here due to the fact that pre-ablation LGE-MRI scans were not obtained. PsAF was defined as sustained AF that lasts >7 days, which is consistent with AHA/ACC/HRS guidelines (Calkins et al., 2017). The approach used to obtain pre-ablation LGE-MGI scans has been described in previous papers (Khurram et al., 2014; Chrispin et al., 2016; Zahid et al., 2016b; Balouch et al., 2017). The Johns Hopkins Institutional Review Board approved, and all patients provided written informed consent for, retrospective study of data collected from these ablation procedures.

### Reconstruction of Patient-Specific Computational Models of the Fibrotic Atria

The approach used to reconstruct atrial models, including patient-specific representations of atrial geometry and fibrotic tissue distribution has been presented in previous papers (McDowell et al., 2012; Roney et al., 2016; Zahid et al., 2016a,b; Deng et al., 2017; Boyle et al., 2018). Briefly, the atrial wall was segmented from MRI scans and LGE and non-LGE regions were delineated using an image intensity ratio approach (Khurram et al., 2014). Three-dimensional finite-element meshes were then constructed for each patient-specific model. These models included a realistic representation of atrial wall thickness. Average edge length ranged from 351.01 to 380.88 μm; mesh size ranged from 1.34 to 2.65 million nodes. Fiber orientations in the atrial myocardium were estimated as described previously (McDowell et al., 2012). We begin with generalized fiber orientations from an atlas human atrial geometry, then use large deformation diffeomorphic metric mapping to morph vectors those onto each patient-specific atrial geometry (Beg et al., 2004; Vadakkumpadan et al., 2009; McDowell et al., 2013). As such, the fiber orientation tensor field is unique in each individual model.

Our methodology for modeling atrial electrophysiology in PsAF patients with fibrotic atria can be found in our published papers (Zahid et al., 2016a,b; Deng et al., 2017; Boyle et al., 2018). Briefly, at the cellular scale in non-fibrotic regions, we used a human chronic AF atrial action potential model (Courtemanche et al., 1998) with modifications to fit clinical monophasic action potential recordings from patients with AF (Krummen et al., 2012). In fibrotic regions, this model was further modified to match relevant experimental data, as described previously (Avila et al., 2007; Corradi et al., 2008; Nattel et al., 2008; Pedrotty et al., 2009; Kakkar and Lee, 2010; Ramos-Mondragon et al., 2011; Zahid et al., 2016a,b). At the tissue scale, as in previous studies (Zahid et al., 2016a,b; Deng et al., 2017; Boyle et al., 2018), conductivity tensor values in both regions were calibrated to match clinical recordings. Briefly, parameters were adjusted in a test slab geometry (4.5 cm × 4.5 cm × 0.5 cm, uniform fiber orientation) to obtain a longitudinal conduction velocity (CV) of 43.39 cm/s, consistent with the range of values measured during clinically mapped AF in humans (38–54 cm/s) (Konings et al., 1994). When the calibrated parameters were used in a test model with patient-specific fiber orientations and regions of fibrotic remodeling, CV values were in the expected range (31.46 [28.38; 36.32] cm/s; min/max: 15.18/47.81 cm/s).

### Identification and Comparison of RD Locations in Simulations and FIRM Data

In each model, rapid pacing was applied at 30 uniformly distributed sites to induce AF. Pacing cycle length was decreased from 300 to 150 ms with the following inter-beat coupling intervals, in ms: 300, 275, 250, 225, 200, 190, 180, 170, 160, 150, and 150. For all 12 stimuli, pulse duration was 5 ms and transmembrane current strength was 0.3 mA/cm^2^. Induced AF episodes were simulated for 2.5 s following the end of pacing. Persistent RD locations observed in simulations (i.e., RD_sim_) were identified by determining phase singularity trajectories (Gray et al., 1998; Eason and Trayanova, 2002), which were extracted using the dynamic wavefront tip trajectory analysis approach (Deng et al., 2017). Briefly, RD_sim_ wavefront “pivot points” were manually identified during a 1,000 ms analysis interval at the end of each simulation. This ensured that multiple RD_sim_ rotations were analyzed and that transient instability immediately following AF initiation was disregarded. In all cases, RD_sim_ persisted for at least two rotations and lasted at least 200 ms, which is consistent with the RD definition of in previous papers (Narayan et al., 2012a; Haissaguerre et al., 2014).

A description of the methodology used to identify RDs from FIRM data (i.e., RD_FIRM_) can be found in previous publications (Chrispin et al., 2016; Balouch et al., 2017). Briefly, a 3D bi-atrial electroanatomical map was constructed with the CARTO system (Biosense Webster) and merged with geometry extracted from pre-ablation MRI scans. AF was induced in patients presenting in sinus rhythm by atrial burst pacing and isoprotenerol infusion. RD_FIRM_ were mapped using a 64-pole basket mapping catheter (FIRMap; Abbott) in both the left and right atria and projected onto the electroanatomical map. This involved the use of proprietary software (RhythmView, Abbott) to derive 2D graphical displays of endocardial activation patterns from basket catheter unipolar electrogram signals.

RD_sim_ and RD_FIRM_ locations were compared on a region-wise basis. Each atrial geometry was manually subdivided into seven anatomically defined regions, as described by Haissaguerre et al. (2014): four regions in the left atrium (LA), two in the right atrium (RA), and one in the interatrial septum. Each RD was classified as belonging to the region in which the majority the RD trajectory was located. Regional classification of RD locations was performed by three different individuals (PB, JH, and MM) who were blinded to each other’s annotations; no discrepancies in classification occurred. As in our previous study (Boyle et al., 2018), each atrial region in each patient was classified into one of four categories: RD_sim_ and RD_FIRM_, RD_sim_ only, RD_FIRM_ only, or no RD activity.

### FIRM-Guided Ablation Protocol and Clinical Follow-Up

The FIRM-guided ablation protocol has also been described in our earlier papers (Chrispin et al., 2016; Balouch et al., 2017). Although all cases in this study were considered retrospectively, we provide a summary of the protocol here to put the acute and long-term outcomes of clinical ablation in context. Standard electrophysiological catheters were advanced to the high RA, his bundle region, and coronary sinus. If the participant was in sinus rhythm, AF was induced by a rapid atrial burst pacing protocol. A 3D mesh of the RA was constructed using the CARTO 3 system (Biosense Webster, Inc., Diamond Bar, CA, United States). Subsequently, a 64-pole basket catheter (50 or 60 mm; Abbott Electrophysiology, Menlo Park, CA, United States) was introduced in the RA. Unipolar electrograms were recorded from the basket catheter at a sampling frequency of 977 Hz and were filtered at 0.05 to 500 Hz (Cardiolab; GE Healthcare, Waukesha, WI, United States). The quality of unipolar electrograms was assessed by the operating physician and adjustments to the catheter position were made to maximize atrial coverage and signal to noise ratio. FIRM mapping was performed with 60 s of unipolar signals collected per epoch. These signals were analyzed using proprietary software (Rhythm View workstation, Abbott, Menlo Park, CA, United States) and RD_FIRM_ were identified as areas of stable rotational activation patterns. Raw basket electrograms were not analyzed in this study. Ablation was performed using a 3.5-mm-tip irrigated catheter (ThermoCool SmartTouch; Biosense Webster, Inc., Diamond Bar, CA, United States) with power at the discretion of the operator (generally 25 W on the posterior wall and 30 W in anterior, septal, and lateral regions for ∼15–30 s at each location). Ablation was continued until abatement of local electrograms. A repeat RD_FIRM_ map was obtained, and any additional identified RD_FIRM_ were ablated. After completion of ablation of RD_FIRM_ located in RA, a transeptal puncture was performed, and the process was repeated in the LA. All observed stable RD_FIRM_ were ablated. In this cohort, focal drivers of AF were not specifically tracked, nor were they targeted for ablation. After completion of the FIRM-guided ablation, PVI was performed using wide area circumferential ablation of the pulmonary vein antra until entrance and exit block was demonstrated for each pulmonary vein. Additional lines were ablated at the attending physician’s discretion.

As described previously (Balouch et al., 2017), routine follow-up including electrocardiographic testing was performed at 3, 6, and 12 months. Additional follow-up for symptomatic patients was performed as needed. Any incidence of AF documented by ECG or a device-recording system lasting ≥30 s, outside of a 3-month post-procedure blanking period, was classified as recurrence.

### Statistics

Continuous variables were expressed as median [IQR] and compared using either the Wilcoxon signed-rank test (for paired comparisons) or the Mann–Whitney test (for unpaired comparisons). After classifying RD_sim_/RD_FIRM_ within anatomical regions, agreement between regions identified by the two modalities was assessed by calculating the modified Cohen’s kappa statistic (*κ*_0_) (Kraemer, 1980). All tests were two-tailed; *p* < 0.05 indicated statistical significance.

## Results

**FIGURE 1:**
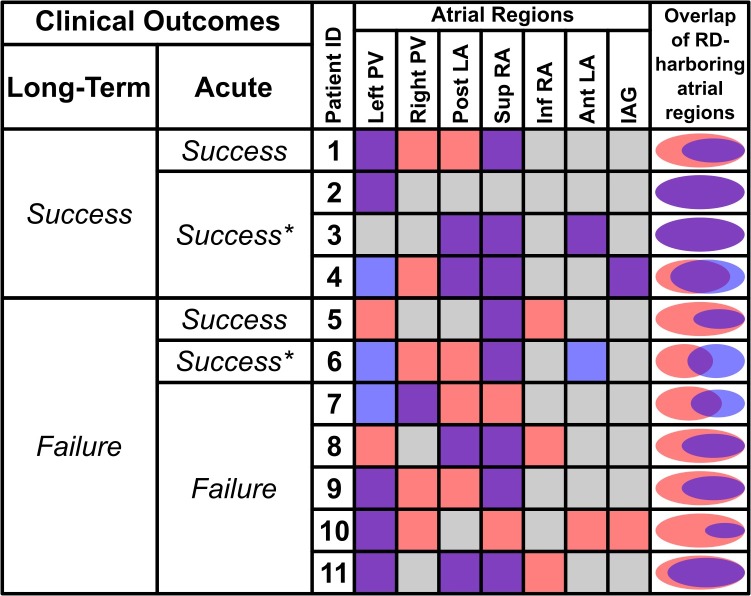
Summary of RD_sim_ and RD_FIRM_-harboring regions for all retrospectively studied individuals and corresponding patient-specific models. The first two columns show long-term and acute outcomes of catheter ablation; see **Table 1** for definition of long-term clinical outcomes. Acute outcomes are defined as Success (AF spontaneously terminated during FIRM-guided to ablation), Success^∗^ (AF organized to atrial flutter during FIRM-guided to ablation), or Failure (AF persisted for the duration of FIRM-guided ablation). Columns under “Atrial Regions” heading show color-coded classification of each part of the atria: *purple* = both RD_sim_ and RD_FIRM_; *gray* = neither RD_sim_ nor RD_FIRM_; *red* = RD_sim_ only; *blue* = RD_FIRM_ only. Rightmost column shows Venn diagrams (to scale) for each patient indicating degree of overlap between RD_sim_- and RD_FIRM_-harboring atrial regions. Data in this figure regarding overlap are based on region-wise comparison (i.e., if a particular atrial region was found to be both RD_sim_-harboring and RD_FIRM_-harboring, that region was deemed to have overlapping RD presence). Here, the concept of overlap is not intended to connote exact physical co-localization of RD_sim_ and RD_FIRM_ sites. PV, pulmonary vein; Post/Ant LA, posterior/anterior left atrium; Sup/Inf RA, superior/inferior right atrium; IAG, inter-atrial groove.

Demographic information about the patient cohort retrospectively analyzed in this study is provided in **Table 1**. No identifiable trends in potential confounding variables (age, sex, BMI, duration of AF prior to ablation, PsAF vs. long-standing PsAF, and proportion of fibrotic tissue as identified by LGE-MRI) were observed. Moreover, there were no differences between success and failure groups in terms of the number of RD_FIRM_ targets ablated or the number of RD_FIRM_-harboring regions. For all 11 individuals, **Figure 1** shows long-term and acute success rates of FIRM-guided ablation procedures (follow-up duration: 230 [180; 326] days), anatomical regions where RD_sim_ and RD_FIRM_ were detected, and Venn diagrams summarizing the degree of overlap between RD_sim_ and RD_FIRM_-harboring regions. As patient IDs were arbitrarily assigned in this retrospective study, we were able to order the patients by long-term outcome then by acute outcome. Detailed information about all 11 clinical procedures, including notes on any ablations other than RD_FIRM_ targets or PVI that were performed, are provided in **Table 2**.

**Table 1:** Demographic and FIRM-guided ablation procedure information for the cohort considered in this study.

Variable	Overall (*n* = 11)	Success (*n* = 4)	Failure (*n* = 7)	*p*-value^∗^
Age	67 [55; 72]	69 [55.5; 72]	67 [55; 69]	0.7485
Male	10 (91)	4 (100)	6 (86)	>0.9999
BMI	29.2 [27.9; 35.3]	32.25 [28.9; 40.48]	28.4 [24.2; 35.3]	0.2515
PsAF	8 (73)	3 (75)	5 (71)	>0.9999
Long-standing PsAF	3 (27)	1 (25)	2 (29)	>0.9999
Duration of AF (y)	4 [2; 10]	2.25 [0.5; 10]	6 [2; 10]	0.3818
Fibrotic tissue (%)	29.1 [18; 47.1]	36.2 [13.73; 46.53]	29.1 [18; 53]	0.6485
RD_FIRM_ targets ablated (#)	3 [2; 6]	4 [2.25; 5.75]	3 [1; 6]	0.5364
RD_FIRM_-harboring regions (#)	2 [1; 3]	2.5 [1.25; 3.75]	2 [1; 3]	0.5788

*Long-term outcomes are defined in terms of freedom from AF during the follow-up period, which was classified as success. ^∗^Comparisons are between patients with different long-term FIRM ablation outcomes.*

**Table 2:** Details of ablation procedures for all 11 patients in the cohort.

Patient ID #	RD_FIRM_ targets ablated (#)	RD_FIRM_-harboring regions (#)	Procedures notes (PVI, other lesions, etc.)
1	3	2	LA roof line
2	2	1	LA posterior roof and floor lines
3	5	3	LA roof line + 2 posterior LA lines
4	6	4	Coronary sinus isolation line
5	1	1	LA roof line
6	6	3	Cavo-tricuspid isthmus ablation (flutter)
7	2	2	LA roof line (flutter)
8	6	2	LA roof line
9	3	2	LA roof line
10	1	1	PVI deferred due to respiratory compensation; FIRM only
11	3	3	No additional lesions

*In 2/11 cases, atypical LA flutter developed after PVI; additional ablation was performed as indicated.*

RD_sim_ were observed in 38 atrial regions (4 [3; 4] per patient); in contrast, RD_FIRM_ were only detected in 24 regions (2 [1; 3] per model; *p* < 0.05 vs. RD_sim_). Classification of atrial regions (as RD-harboring or not) was in agreement between simulations and FIRM more frequently than it differed (5 [4; 6] vs. 2 [1; 3], *p* < 0.05, see **Table 3**). Analysis of inter-rater agreement yielded *κ*_0_ = 0.323, which indicates a moderate degree of consensus (Kraemer, 1980). These findings are consistent with our expectation that RD_sim_ and RD_FIRM_ locations would only partially agree due to the fact that FIRM is capable of identifying only the specific RDs that are manifest during the procedure, whereas simulations can predict all potential RDs arising from the fibrotic substrate. Indeed, the majority of cases where regional classification differed (18/22; 82%) involved latent RD sites in regions where such activity was never detected during FIRM.

**Table 3:** For each patient model, number of atrial regions that were identified as RD_sim_-harboring, RD_FIRM_-harboring, both, or neither.

	# Regions that agreed			# Regions that differed		
Patient ID #	Both RD_sim_ and RD_FIRM_	Neither RD_sim_ nor RD_FIRM_	Total	RD_sim_ only	RD_FIRM_ only	Total
1	2	3	5	2	0	2
2	1	6	7	0	0	0
3	3	4	7	0	0	0
4	3	2	5	1	1	2
5	1	4	5	2	0	2
6	1	2	3	2	2	4
7	1	3	4	2	1	3
8	2	3	5	2	0	2
9	2	3	5	2	0	2
10	1	2	3	4	0	4
11	3	3	6	1	0	1
Total	20	35	55	18	4	22
Median [IQR]	2 [1; 3]	3 [2; 4]	5 [4; 6]	2 [1; 2]	0 [0; 1]	2 [1; 3]

**FIGURE 2:**
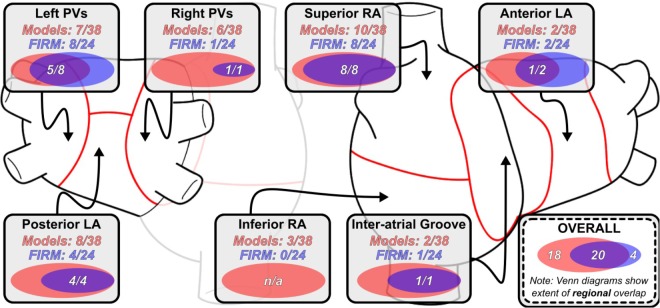
Regional distribution of RD_sim_ and RD_FIRM_-harboring regions. Venn diagrams (to scale) for indicate the degree of overlap between RD_sim_- and RD_FIRM_ in that particular region across all 11 patients. Overlaid numbers indicate the number of clinically observed RD_FIRM_-harboring regions that were correctly reproduced in simulations conducted in the corresponding patient-specific models. As in **Figure 1**, data in this figure regarding overlap are based on region-wise comparison and do not necessarily connote exact physical co-localization of RD_sim_ and RD_FIRM_ sites. See **Figure 1** for expansion of abbreviations.

Distribution of RD_sim_ and RD_FIRM_-harboring regions is summarized in **Figure 2**. The most common sites of RD_sim_ occurrence (31/38; 82%) were the left and right PVs, the posterior LA, and the superior RA; in contrast, the majority of RD_FIRM_ were observed in the left PVs and the superior RA (16/24; 67%). This observation is reinforced by the fact that these two regions were the most likely to harbor latent RD sites (right PVs: 5/18, 28%; posterior LA: 4/18, 22%).

**FIGURE 3:**
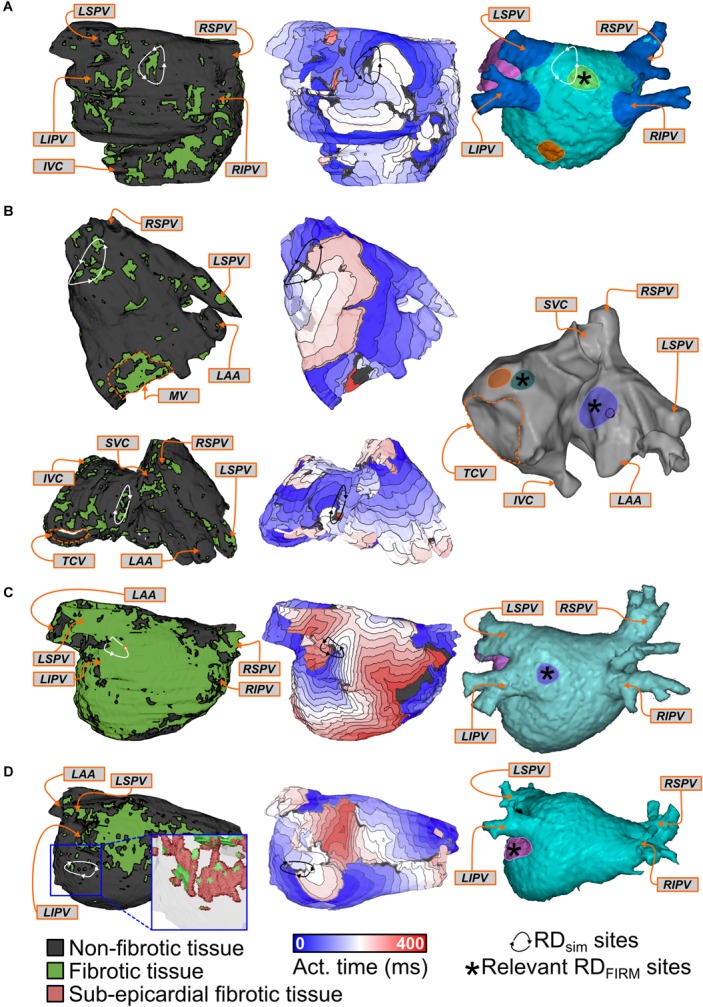
Examples showing direct evidence of co-localization of RD_sim_ and RD_FIRM_. Each row shows the distribution of fibrotic tissue in the patient-specific model (*left*), with anatomical labels (*orange boxes* and *arrows*) for ease of navigation; an activation map highlighting the location of an RD_sim_ that perpetuated AF during simulations (*middle*); and an annotated map exported from the electro-anatomical mapping system (*right*). *Black* regions indicate tissue that did not activate during the relevant interval; locations of RD_FIRM_ are highlighted by brightly colored regions. Some variability existed in the visual due to differences in the export process that could not be made consistent across all 11 cases. **(A)** Patient 8: Matching RD_sim_ and RD_FIRM_ (*yellow*) sites in the posterior LA region. Note that the figure also shows a second, more inferior posterior LA target (*orange*). The PVs and LA appendage were colored *blue* and *pink* for ease of clinical navigation (i.e., there were no RD_FIRM_ in these colored regions). **(B)** Patient 4: Matching RD_sim_ and RD_FIRM_ sites in the inter-atrial groove (FIRM annotation: *blue*) and superior RA (FIRM annotation: *green*) regions of the atria. A third RD_FIRM_ is shown deeper inside the RA appendage (*orange*). **(C)** Patient 10: Matching RD_sim_ and RD_FIRM_ (*blue*) sites in the left PV region. The LA appendage was colored *pink* for ease of clinical navigation. **(D)** Patient 9: Matching RD_sim_ and RD_FIRM_ (*pink*) sites in the left PV region. Inset: View of RD_sim_ region with non-fibrotic tissue rendered as semi-transparent to show 3D transmural distribution of fibrotic tissue, including extensive sub-epicardial fibrotic remodeling in this area (*red*).

Side-by-side visualizations of RD_sim_ and RD_FIRM_ sites for four different patients are shown in **Figure 3**. In each case, the spatial distribution of fibrotic tissue in the same model is also included. For Patient 8 (**Figure 3A**), the example shown is for an RD_sim_ located in the posterior LA, roughly at the center of the plane formed by the four pulmonary veins; RD_FIRM_ was documented for the same patient at a similar location during FIRM (yellow highlighted region). For Patient 4 (**Figure 3B**), two examples are shown. First (top row), an RD_sim_ in the anterior part of the inter-atrial groove region, inferior to the right superior PV; second (bottom row), an RD_sim_ in the superior RA, near the base of the RA appendage. These sites correspond, respectively, to the blue and green highlighted regions in the FIRM schematic shown. For Patient 10 (**Figure 3C**), the case shown is for an RD_sim_ near the left PVs, which corresponded to a FIRM-mapped site (blue). Finally, for Patient 9 (**Figure 3D**), the highlighted RD_sim_ is found in the left PV region, between the left inferior PV and the mitral valve annulus, which is a direct match to the RD_FIRM_ area (pink). Notably, although this particular RD trajectory persists within a region that appears non-fibrotic from the epicardial surface, the underlying transmural and endocardial tissue in that area is quite fibrotic (see inset).

**FIGURE 4:**
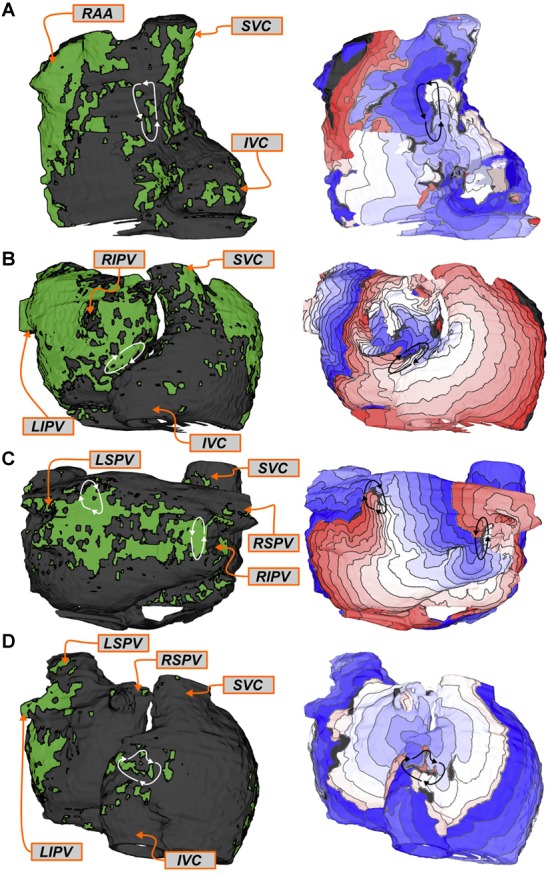
Examples of regions that harbored RD_sim_ only (i.e., latent RD_sim_). See **Figure 3** for legends. **(A**,**B)** Patient 10: Latent RD_sim_ sites in the inter-atrial groove **(A)** and right PV **(B)** regions. **(C)** Patient 9: Latent RD_sim_ in the posterior LA (*upper left*) and right PV (*right*) regions; see text for further discussion of this unique case in which two RD_sim_ simultaneously persisted during a single simulated AF episode. **(D)** Patient 5: Latent RD_sim_ in the inferior RA.

Four examples of latent RD_sim_ sites (i.e., located in regions that were not classified as RD_FIRM_-harboring) are shown in **Figure 4**. Two separate AF episodes are shown for Patient 10, first for an RD_sim_ observed in the inter-atrial groove region on the anterior side of the RA near the superior vena cava (**Figure 4A**); second, for an RD_sim_ in the right PV region, near the carina between the right superior and inferior PVs (**Figure 4B**). For Patient 9, the example shown (**Figure 4C**) highlights simulated AF that was perpetuated by two simultaneous RD_sim_, one very close to the right inferior PV and the other on the posterior LA, toward the left superior PV. This was the only case in which this particular dynamic (i.e., more than one stable RD_sim_ persisting simultaneously) was observed in this study; for purposes of RD_sim_ classification, this example led to both the right PV and posterior LA regions being annotated as RD_sim_-harboring. Finally, for Patient 5, the RD_sim_ shown is in the inferior RA on the posterior side of the inferior vena cava (**Figure 4D**). In all four examples presented above, RD_FIRM_ activity was not documented in the given atrial region during the clinical ablation procedure.

**FIGURE 5:**
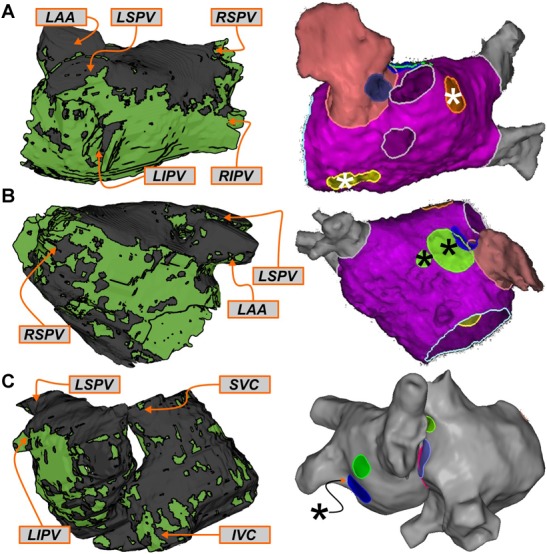
Examples of regions that harbored RD_FIRM_ only. See **Figure 3** for legends. **(A**,**B)** Patient 6: Clinically mapped RD_FIRM_ sites in the left PV (**A**; *yellow/orange* highlights) and anterior LA (**B**; *green* highlights) regions, within which RD_sim_ were never observed in the corresponding model. **(C)** Patient 4: RD_FIRM_ region in the left PV region (*blue* highlight). In both cases, several other mapped RD_FIRM_ sites (not relevant to this specific figure) are shown in the FIRM schematics, since it was not possible to export these images without all of the clinical annotations.

For this cohort, most of atrial regions in which RD_FIRM_ were observed were also found to be RD_sim_-harboring during simulation analysis (20/24; 83%). Three examples of RD_FIRM_ that were not observed in the corresponding models are shown in **Figure 5**. For Patient 6, two instances are presented: first, two RD_FIRM_ in the left PV region (yellow and orange highlighted areas in **Figure 5A**); second, another two RD_FIRM_ on the anterior LA, at the base of the LA appendage (green highlighted areas in **Figure 5B**). For Patient 4, an RD_FIRM_ was identified in the left PV region (blue highlighted area in **Figure 5C**). In all of these cases, RD_sim_ were not observed in the corresponding regions of the patient-specific model. Another possibility that cannot be excluded is that these RD_FIRM_ sites are false positives.

**FIGURE 6:**
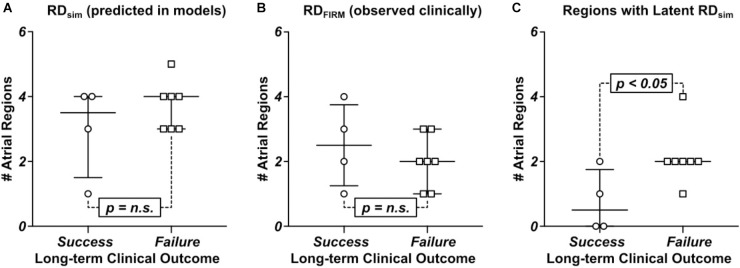
Comparison of numbers of RD_sim_ and RD_FIRM_-harboring regions between patients whose catheter ablation procedure succeeded or failed long-term. See **Table 1** for clinical definition of long-term ablation outcomes. **(A)** RD_sim_-harboring regions predicted in models. **(B)** RD_FIRM_-harboring regions observed clinically. **(C)** Regions that harbored latent RD_sim_.

In general, the number of RD_sim_-harboring regions observed in computational models differed significantly between those reconstructed from MRI scans of patients who did not experience AF recurrence during follow-up vs. those in whom the procedure was classified as a long-term failure. There was a trend toward more RD_sim_-harboring regions in the seven patients whose ablation failed compared to the four individuals in whom the procedure succeeded (**Figure 6A**; failure: 4 [3; 4] vs. success: 3.5 [1.5; 4], *p* = n.s.). Interestingly, this trend was reversed for RD_FIRM_-harboring regions (**Figure 6B**; failure: 2 [1; 3] vs. success: 2.5 [1.25; 3.75], *p* = n.s.), although neither difference rose to the level of significance. In the four patients who had long-term freedom from AF, there were only three examples of latent RD_sim_ sites in two patients; in contrast, all patients who had recurrent AF during follow-up had at least one latent RD_sim_ site (**Figure 6C**; failure: 2 [2; 2] vs. 0.5 [0; 1.75], *p* < 0.05).

## Discussion

Our study has important implications for understanding of the fibrotic substrate for arrhythmia initiation and perpetuation in PsAF patients. Nearly all of the atrial regions (84%) where RD_FIRM_ were observed also harbored RD_sim_ in simulations, which reinforces the validity of our patient-specific modeling technique and supports the notion that many rotors mapped during clinical procedures are perpetuated by the fibrotic substrate. Interestingly, in the subset of patients (*n* = 7) in whom the long-term outcome of ablation was failure (AF recurred during follow-up), we uncovered a large number of additional RD_sim_-harboring regions (2 [2; 2] per individual), within which rotor activity was never identified by FIRM. These locations, termed latent RD_sim_ sites, were significantly less prevalent in models corresponding to patients who remained free from AF for the duration of follow-up (*n* = 4; 0.5 [0; 1.75] per individual). These results suggest that one potential explanation for the failure of FIRM-guided ablation is the prevalence of latent RD_sim_ sites. It also suggests that ablation procedures based on custom-tailored plans to exhaustively target all potential RD_sim_ locations could lead to better long-term outcomes for PsAF patients.

Another interesting finding of our study was that the prevalence of latent RD_sim_ sites was particularly high in two specific atrial regions: the posterior LA and the right PVs. There are several potential explanations for this finding. On one hand, due to inter-patient variability in atrial geometry and fibrotic tissue distribution, it is possible that many individuals in this cohort happened to have the necessary substrate for rotor perpetuation in those regions but re-entrant activity did not happen to manifest there during mapped AF episodes. On the other hand, it is also conceivable that rotors *did* manifest in those atrial regions in some or all of the apparent latent RD_sim_ sites, but FIRM failed to uncover them as RD_FIRM_. If the latter is the case, it is also unclear whether this is due to a shortcoming in the FIRM system’s ability to record rotors in these regions or some other factor (e.g., the specific way the basket catheter was deployed during the ablation procedures in question). Finally, since there is no gold standard for evaluating rotor presence during AF episodes or confirming their causative role in arrhythmia perpetuation, we cannot formally exclude the possibility that the prevalence of latent RD_sim_ sites is partly or wholly attributable to false positives in simulations; however, the fact that they were observed more frequently in models corresponding to patients who experienced AF recurrence during follow-up provides modest correlative evidence that this is not the case.

Previous studies, both from our group (Boyle et al., 2018) and others (Tanaka et al., 2007), have pointed to the left PV and posterior LA regions as the most likely to harbor rotors. For the patient-specific models considered in this study, we also observed numerous RD_sim_ in those regions (left PVs: 7/38 [18%]; posterior LA: 8/38 [21%]) but RD_sim_ were just as likely to manifest in the right PV and superior RA regions (6/38 [16%] and 10/38 [26%], respectively). This highlights the importance of attempting to map AF-perpetuating activity in the RA in addition to the LA, since many putative targets (in both simulations and FIRM) were identified in the upper part of that chamber. Notably, in three of the four cases where the procedure resulted in long-term freedom from AF (see **Figure 1**), RD_FIRM_ were observed and ablated in areas that would not be targeted as part of a PVI procedure (superior RA, anterior LA, and/or inter-atrial groove), regardless of whether roof or floor lines were added in addition to standard circumferential lesions in the PV antra. This reinforces the general value of substrate-based ablation in the PsAF population.

In our earlier study (Boyle et al., 2018), which compared rotors detected by pre-ablation non-invasive ECGI to those predicted by simulations, the majority of atrial regions where the two modalities differed (23/32 [72%]) were cases where an RD_sim_ was not observed in the model even though it was recorded during mapped AF. In contrast, for the present study, the number of RD_FIRM_-only regions was small (only four instances across all patients), meaning that the differences between FIRM and simulations were dominated by latent RD_sim_ sites. Previously, we primarily attributed the prevalence of rotors detected by ECGI but not predicted by simulations to rotors perpetuated by mechanisms other than the fibrotic substrate, but the present finding raises an intriguing alternative explanation. Namely, rotor detection via ECGI may be susceptible to a high rate of false positives (i.e., incorrect classification of non-rotor activity as an ablation targets), especially compared to RD_FIRM_ detection, which our findings conversely suggest is vulnerable to false *negatives* (i.e., the failure to observe latent RD_sim_ sites). This putative over-sensitivity of rotor detection via ECGI would also explain why the incidence of latent RD_sim_ in our previous study was low. However, a more systematic study involving rotor identification via all three methods in the same cohort, would be necessary in order to test this hypothesis. The development of a definitive gold standard for rotor identification would also be helpful in advancing knowledge in this area.

Finally, our discovery that latent RD_sim_ sites existed in all seven patients who had failed FIRM-guided ablation procedures but only two of the four individuals who benefited from treatment could explain why some recent trials have reported limited efficacy of PVI + FIRM compared to PVI alone (Mohanty et al., 2018). However, the size of the cohort in our study is quite small (*n* = 11) and the number of patients who did not experience recurrent AF was even smaller (*n* = 4), thus it is difficult for us to draw definitive conclusions. Moreover, due to the retrospective nature of our study, the data presented here cannot definitively prove that latent RD_sim_ sites were the underlying cause of AF recurrence. Future work will need to test this hypothesis by determining prospectively if clinical ablation of all potential rotor sites, as identified by simulations conducted in patient-specific models of the fibrotic atria or by some other means, can deliver robust and long-term freedom from AF in PsAF patients.

As outlined in Section “Materials and Methods,” our model reconstruction approach uses a complex image-based technique to morph human atrial atlas fiber orientations onto each patient-specific geometry to create a unique conductivity tensor field. This is the most advanced approach currently undertaken, given clinical MRI has not yet developed to the point where it would be feasible to discern patient-specific fiber orientations or fine-grain structural details (e.g., endocardial bundles). Complexities of the atrial endocardial microstructure have recently been shown to influence RD localization in experiments and simulations conducted in models reconstructed from high-resolution *ex vivo* MRI scans (Hansen et al., 2015; Zhao et al., 2017). The data presented here provide important insights based on dynamic localization of RD_sim_ arising from the macroscopic distribution of fibrotic and non-fibrotic tissue (which can be obtained non-invasively via standard clinical imaging techniques), however, we cannot exclude the possibility that these mechanisms might interact with other potential factors (e.g., variability in myocardial thickness, endocardial bundles, etc.) to give rise to the complex behavior of AF-perpetuating rotors in patients.

The FIRM approach is capable of identifying both reentrant (i.e., RD_FIRM_) and focal AF drivers. However, for the patient cohort examined in the present study, the incidence of focal drivers was not tracked. Since study was retrospective, we cannot offer any insights on the debate regarding the relative importance of focal vs. RDs. Moreover, since our computational approach is specifically designed to identify regions within each patient’s unique fibrotic substrate where RD_sim_ could potentially be sustained, it is not suitable for investigating the role(s) played by focal drivers in perpetuating AF.

Finally, a limitation of this project is that due to the retrospective nature of the study, we cannot evaluate the physical distance between RD_FIRM_ and RD_sim_ sites, as CARTO maps from FIRM ablation procedures could not be co-registered with model geometry reconstructed from LGE-MRI. Because of that, rather than comparing specific RD_FIRM_ and RD_sim_ locations, we instead opted to compare RD_FIRM_- and RD_sim_-harboring *regions*, as done in previous work by us and others (Haissaguerre et al., 2014; Boyle et al., 2018), with the acknowledgment that these atrial regions are relatively large.

## Conclusion

The presence of numerous atrial regions in multiple patients that were found to harbor both RD_FIRM_ and RD_sim_ reinforces our previous finding that PsAF is partly driven by fibrosis-mediated mechanisms and supports the validity of our computational modeling approach. Our analysis highlights the prevalence of latent RD_sim_ sites as a potential explanation for failed ablation procedures in PsAF patients. Finally, we show that long-term outcomes were better in patients where there were fewer latent RD_sim_ sites, suggesting that substrate-based ablation based on computational modeling may be able to identify a more exhaustive set of PsAF substrate ablation targets compared to intracardiac mapping.

## Author Contributions

PB, SZ, SN, DS, and NT conceived the idea. PB, JH, SZ, WF, and MM developed the patient-specific atrial models, ran the computational simulations, and analyzed the outputs thereof. PB, JH, SZ, WF, MM, AP, KA, TZ, MB, EI, JC, DS, and NT interpreted the imaging data, FIRM-guided ablation, and simulation results. JC, RB, HA, JM, HC, SN, and DS oversaw and carried out the FIRM-guided ablation procedures. PB and NT wrote the manuscript.

## Conflict of Interest Statement

The authors declare that the research was conducted in the absence of any commercial or financial relationships that could be construed as a potential conflict of interest.
